# Nucleation mechanism of nano-sized NaZn_13_-type and α-(Fe,Si) phases in La-Fe-Si alloys during rapid solidification

**DOI:** 10.1186/s11671-015-0843-1

**Published:** 2015-03-19

**Authors:** Xue-Ling Hou, Yun Xue, Chun-Yu Liu, Hui Xu, Ning Han, Chun-Wei Ma, Manh-Huong Phan

**Affiliations:** Laboratory for Microstructures, Shanghai University, Shanghai, 200444 China; School of Materials Science and Engineering, Shanghai University, Shanghai, 200072 China; Shanghai University of Engineering Science, Shanghai, 201620 China; Department of Physics, University of South Florida, Tampa, FL 33620 USA

**Keywords:** La(Fe,Si)_13_ ribbons, Nucleation mechanism, Nanostructure, Rapid solidification

## Abstract

The nucleation mechanism involving rapid solidification of undercooled La-Fe-Si melts has been studied experimentally and theoretically. The classical nucleation theory-based simulations show a competitive nucleation process between the α-(Fe,Si) phase (size approximately 10 to 30 nm) and the cubic NaZn_13_-type phase (hereinafter 1:13 phase, size approximately 200 to 400 nm) during rapid solidification, and that the undercooled temperature change ∆*T* plays an important factor in this process. The simulated results about the nucleation rates of the α-(Fe,Si) and 1:13 phases in La-Fe-Si ribbons fabricated by a melt-spinner using a copper wheel with a surface speed of 35 m/s agree well with the XRD, SEM, and TEM studies of the phase structure and microstructure of the ribbons. Our study paves the way for designing novel La-Fe-Si materials for a wide range of technological applications.

## Background

La-Fe-Si alloys exhibiting a giant magnetocaloric effect (GMCE) near room temperature are one of the most promising candidate materials for advanced magnetic refrigeration technology [1-4]. In La-Fe-Si alloys, the NaZn_13_-type phase (1:13 phase), which undergoes a first-order magneto-structural transition accompanied by a typical itinerant electron metamagnetic transition and a large volume change in the vicinity of its Curie temperature *T*_C_, has been reported to be a driving force for achieving the GMCE [5-8]. From a materials perspective, the 1:13 phase with a cubic NaZn_13_-type (Fm $\overline{3}$ c) structure is very difficult to form directly from equilibrium solidification conditions. It has been shown that during an equilibrium solidification, α-(Fe,Si) phase (A2: Im $\overline{3}$ m) dendrites firstly grow from the liquid as the primary phase and then a peritectic reaction with the surrounding liquid occurs to form the 1:13 phase (α-(Fe,Si) + L → 1:13 phase). Trace amounts of a La-rich or a LaFeSi phase are also found in the interdendritic region [9,10]. It is a major difficulty to produce the 1:13 phase because of its low phase stability at elevated temperatures and low atomic diffusivity [11,12]. Due to the incompleteness of the peritectic reaction, a large number of α-(Fe,Si) dendrites are preserved at room temperature. In as-cast conditions attained by conventional arc-melting techniques, La-Fe-Si alloys show a two-phase structure composed of α-(Fe,Si) and La-Fe-Si (Cu_2_Sb-type: P4/nmm) phases. It is therefore essential to anneal the as-cast alloys in vacuum at a high temperature for a long time (approximately 1,323 K, 30 days) to gain the desired 1:13 phase. Recently, the melt spinning technique has emerged as a more efficient approach for producing La(Fe,Si)_13_ materials, since the desired 1:13 phase could be obtained subject to a much shorter time annealing (approximately 1,273 K, 20 to 120 min) [11,12]. The primary contents of α-(Fe,Si) and 1:13 phases obtained from the melt spinning technique are entirely different from those obtained using conventional equilibrium solidification techniques [13]. However, the origin of this difference has remained an open question. While the nucleation mechanism of 1:13 and α-(Fe,Si) phases in La-Fe-Si alloys during rapid solidification has been yet investigated, knowledge of which is key to exploiting their desirable properties for a wide range of technological applications.

To address these emerging and important issues in the present work, we have investigated theoretically and experimentally the nucleation mechanism of α-(Fe,Si) and 1:13 phases in melt-spun La-Fe-Si ribbons. Detailed microstructural studies of the wheel-side and free-side surfaces of the melt-spun ribbons are reported. Our simulated and experimental results consistently show that there exists a competitive nucleation process between the nano-sized α-(Fe,Si) and 1:13 phases during rapid solidification, and that the undercooled temperature change, ∆T, plays a crucial factor in this process. A similar trend has also been reported in other peritectic alloys [14-17].

## Methods

Button ingots with a nominal composition of LaFe_11.5_Si_1.5_ were prepared by arc-melting 99% La, 99.9% Fe, and 99.5% Si crystals in an argon gas atmosphere. The ingots were remelted four times and each time the button was turned over to obtain a homogeneous composition. The button was broken into pieces, and these pieces were then put into a quartz tube with a nozzle. The chamber of the quartz tube was evacuated to a vacuum of 3 to 5 × 10^−3^ Pa and then filled with high-purity Ar. The samples were melted by electromagnetic induction and then ejected through the nozzle using a pressure difference into a turning cooper wheel. The surface speed of the Cu wheel was approximately 35 m/s to get ribbon samples with a thickness about 25 μm. Here, we denote the surface of the ribbon far from the copper wheel as the free surface, while the surface of the ribbon in direct contact with the copper wheel is referred to as the cooled surface. The phases and crystal structures of the ribbons were characterized by powder X-ray diffraction (XRD) using Cu-K_α_ radiation. The microstructure analysis was carried out by a scanning electron microscope (SEM) with an energy dispersive spectrometer (EDS) (model JSM-6700 F, JEOL Ltd., Tokyo, Japan) and a transmission electron microscope (TEM, model JEM-2010 F, JEOL Ltd., Tokyo, Japan). The TEM specimen was prepared by a dual-beam focused ion beam (FIB, model 600 i, FEI Company, Oregon, USA).

## Results and discussion

**Figure 1:**
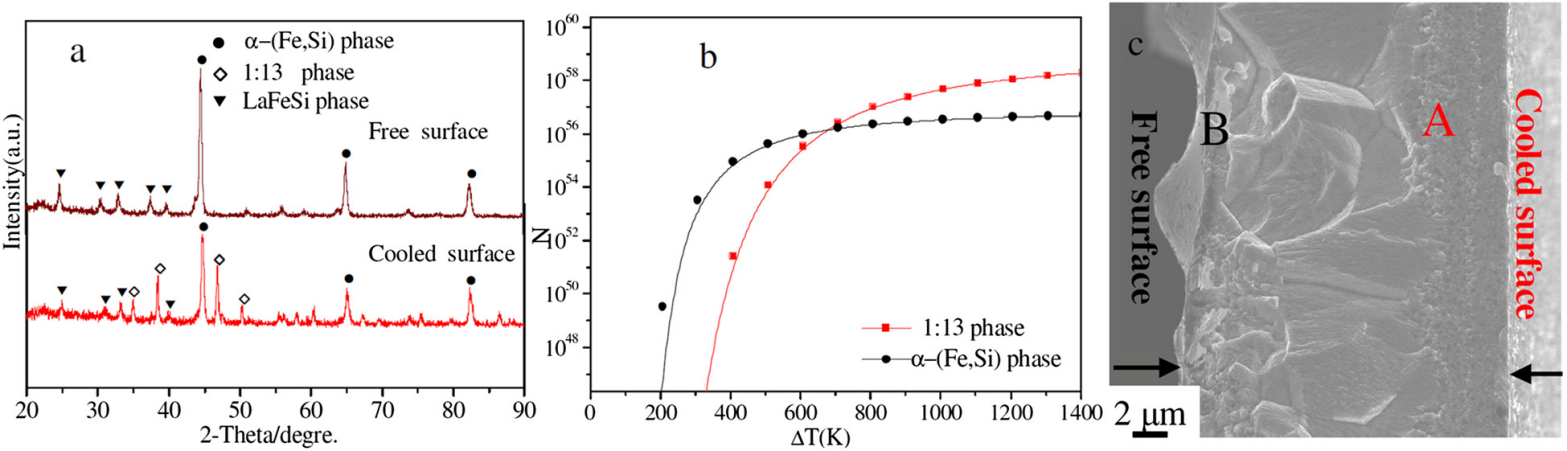
**The room temperature XRD patterns, SEM, and simulated results using the classical nucleation theory. (a)** XRD patterns of both the cooled and free surfaces of a melt-spun La-Fe-Si ribbon; **(b)** nucleation rates of α-(Fe,Si) and La(Fe,Si)_13_ phases versus La-Fe-Si alloy; and **(c)** a cross-sectional SEM image of the ribbon indicating the microstructural difference between the cooled surface region (region A) and the free surface region (region B).

**Figure 2:**
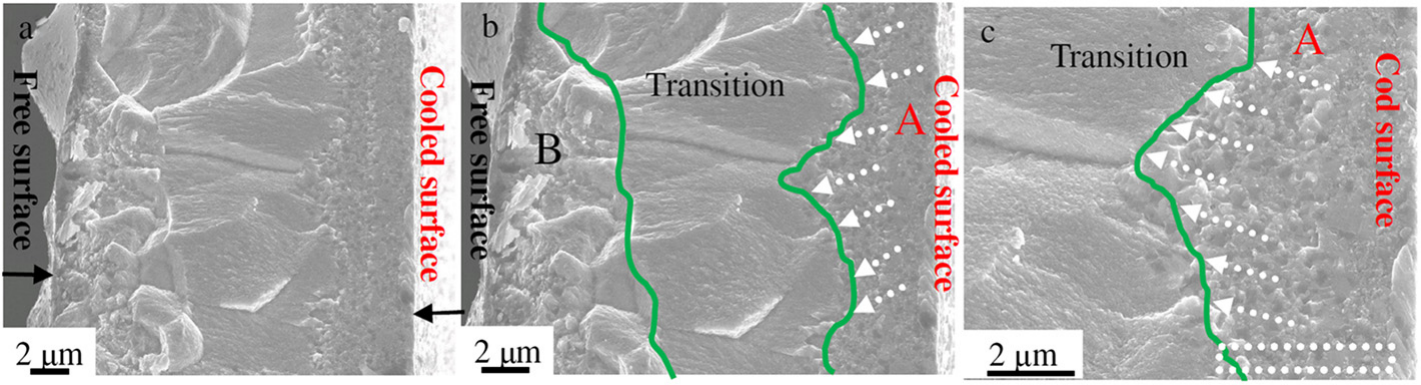
**Cross-sectional SEM images.** Cross-sectional SEM images of a melt-spun La-Fe-Si ribbon **(a)** with different magnifications **(b,c).** There exist three different regions: region A (the cooled surface), region B (the free surface), and a transitional region between A and B.

The room temperature XRD patterns, SEM, and simulated results using the classical nucleation theory, as shown in Figure 1a and c, reveal the change in composition and volume fraction of the α-(Fe,Si) and 1:13 phases on the cooled surface and free surfaces of a melt-spun ribbon. As one can see clearly in Figure 1a, the XRD patterns show that the majority of the 1:13 phase is on the cooled surface of the ribbon, while this phase diminishes, even disappears, when crossing toward the other surface of the ribbon. The majority of the α-(Fe,Si) phase is found on the free surface. The as-cast microstructure appears to be very different between the cooled and free surfaces of the ribbon (see regions A and B of Figure 1c). These results indicate that the rapid solidification process favors a direct formation of the 1:13 phase from the liquid melt of La-Fe-Si. By contrast, under an equilibrium solidification condition, the 1:13 phase is formed via a peritectic reaction process between the nascent α-(Fe,Si) and liquid (L) phase (1:13 → α-(Fe,Si) + L). It is worth noting that there is a distinct difference in the formed phase structure and microstructure between the cooled and free surfaces of the melt-spun ribbon. This can be attributed to the difference in the nucleation rates of the α-(Fe,Si) and 1:13 phases. According to the classical nucleation theory (CNT) [18,19], the heterogeneous nucleation rate can be determined by

1 $$ I=\frac{k_{\mathrm{B}}T{N}_{\mathrm{n}}}{3\pi \upeta (T){a}_o^3}. exp\left[-\frac{\varDelta G*}{k_{\mathrm{B}}T}\right], $$

where *k*_B_, η(*T*), *N*_n_, *a*_0_, and ∆G* are the Boltzmann constant, the temperature-dependent viscosity of the undercooled melt, the potential nucleation sites, the average atomic distance, and the activation energy for forming a critical nucleus, respectively. ∆G* cas

2 $$ \varDelta {G}^{*}=\frac{16\pi }{3}\frac{\sigma^3}{\varDelta {G}_{\mathrm{V}}^2}f\left(\theta \right)=\frac{16}{3}\frac{\sigma^3\varDelta {S}_{\mathrm{f}}{T}_{\mathrm{l}}^3}{{\left({T}_{\mathrm{l}}-T\right)}^2}f\left(\theta \right), $$

where σ, ∆*G*_v_, ∆*S*_f_, *T*_l_, *T*, and *f*(θ) are the interfacial energy, the Gibbs free energy difference between liquid and solid, the entropy of fusion, the liquid temperature, the temperature, and the catalytic factor for nucleation, respectively. The interfacial energy, σ, can be estimated by the model developed by Spaepen [19,20]:

3 $$ \sigma =\alpha \frac{\varDelta {S}_{\mathrm{f}}T}{{\left({N}_1{V}_{\mathrm{m}}^2\right)}^{1/3},} $$

where α is the structure-dependent factor, *N*_l_ is Avogadro constant, and *V*_m_ is the volume. By inserting the essential parameters listed in Table 1 into Eqs. 1 to 3, the heterogeneous nucleation rates for the α-(Fe,Si) and 1:13 phases can be simulated. The calculated results (Figure 1b) show a competing nucleation between the α-(Fe,Si) and 1:13 phases that occurred during rapid solidification. As the undercooled temperature change, ∆*T*, is less than 707 K, the α-(Fe,Si) phase has a higher nucleation rate compared to that of the 1:13 phase on the free surface of the ribbon, and it is therefore a primary phase in a slow solidification process. For ∆*T* > 707 K, however, the reverse situation is observed. The nucleation rate of the 1:13 phase is faster than that of the α-(Fe,Si) phase on the cooled surface of the ribbon, thus resulting in the 1:13 phase as a primary solidification phase. These calculated results are well interpreted from the obtained XRD data (Figure 1a). When ∆*T* = 707 K, the nucleation rate of the 1:13 phase is equal to that of the α-(Fe,Si) phase. This is seen as an intersection of the two curves of Figure 1b, where the microstructure is found to be an obvious watershed between the cooled and free surfaces of the ribbon (see Figure 1c and Figure 2a); this watershed matches with the green lines of Figure 2b and c. It can also be seen in Figure 1c that small-sized grains in region A are on the cooled surface, and its microstructure is very different from that of region B of the free surface. The ‘transition’ region from region A (the cooled surface) to region B (the free surface) can be seen in cross-sectional SEM images with higher magnifications (Figure 2b and c), where the small-sized grains appeared as rectangular black dots in region A (Figure 2c). The chemical compositions were observed to change between regions A and B during the rapid solidification process of La-Fe-Si. EDS analysis showed that the content of La and Si in region A was higher than those of region B and of the nominal composition (see Table 2). The content of Fe was lower than those of region B and of the nominal composition. The variations in the chemical compositions in regions A and B are likely associated with the varying contents of α-(Fe,Si) phase.

**Table 1:** **Physical parameters of the La-Fe-Si alloy** [21] **used in our calculations**

**Parameters**	**α**-**(Fe,Si) phase**	**1:13 phase**
*k*_B_ (J/K)	1.38 × 10^−23^	1.38 × 10^−23^
*N*_l_	6.02 × 10^23^	6.02 × 10^23^
f(θ)	0.3	0.3
*T*_L_ (K)	1,811	1,698
*a*_0_ (m)	2.48 × 10^−10^	1.56 × 10^−12^
*V*_m_ (m^3^°mol^−1^)	7.1 × 10^−6^	8.1 × 10^−6^
α	0.71	0.417
*N*_n_ (J/mol K)	1.572 × 10^25^	1.572 × 10^25^
σ	0.350	0.430
η (T)	0.09	0.09
Δ*S*_f_ (J/mol K)	8.48	20.67
ΔG*	$\frac{1.09\times {10}^{10}}{{\left(\varDelta T\right)}^2}$	$\frac{4.04\times {10}^{10}}{{\left(\varDelta T\right)}^2}$
Δ*G*_V_	$\frac{2.16\times {10}^9}{{\left(\varDelta T\right)}^2}$	$\frac{8.05\times {10}^9}{{\left(\varDelta T\right)}^2}$

**Table 2:** **The chemical compositions determined by EDS for regions A and B of the melt-spun La-Fe-Si ribbon (Figure** 2 **) relative to its nominal composition**

**Region**	**La (at.%)**	**Fe (at.%)**	**Si (at.%)**
A	8.80	79.45	11.53
B	8.29	85.05	6.67
Nominal composition	8.35	81.2	10.82

Note that we added a 5% burning loss in La element because it was easy to volatile during the melting process of La-Fe-Si.

**Figure 3:**
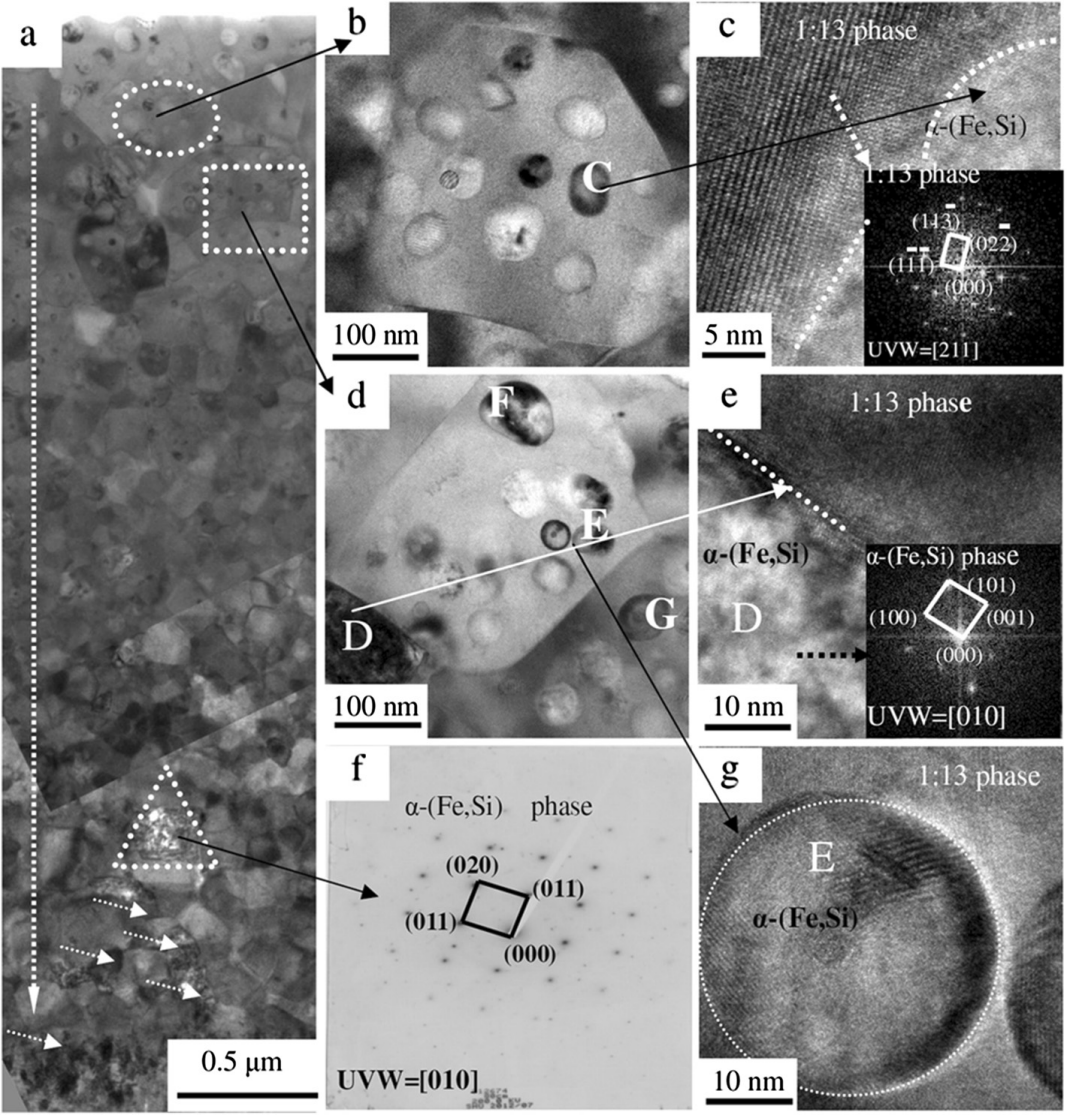
**The global microstructural morphology.** A cross-sectional TEM micrograph of the cooled surface region (region A) **(a)**, with higher magnifications of the regions of the sample indicated by the dashed circle **(b)** and box **(d)**. HRTEM micrographs for regions C, D and E and its corresponding FFT patterns in **(c)**, **(e)** and **(g)**; SAED pattern of the ‘dashed triangle’ of Figure 4a is shown in Figure 4 **(f)**.

Figure 3a shows the global microstructural morphology of the cooled surface of the ribbon for region A. Expanded views of the white circle and square areas of Figure 3a are shown in Figures 3b and d, respectively. The HRTEM images and corresponding Fourier transforms for ‘C’ of Figure 3b, a large dark spherical precipitate region and its adjacent matrix, are displayed in Figure 3c, where the matrix is indexed to the structure of the 1:13 phase, while the spherical precipitate ‘C’ is indexed to the α-(Fe,Si) phase with approximately 97.26 at.% Fe and 2.74 at.% Si as determined by EDS. Using the same analysis, the spherical precipitates ‘E’, ‘F’, and ‘G’ in Figure 3d are determined to be the α-(Fe,Si) phase with the chemical compositions of approximately 96 to 98 at.% Fe and 4 to 2 at.% Si. It can be observed that the Moire fringes in Figure 3 g are two adjacent spherical precipitates of α-(Fe,Si) in ‘E’ of Figure 3d. These spherical α-(Fe,Si) phases are embedded in the 1:13 matrix. The sheet labeled ‘D’, with an adjacent 1:13 matrix, can be indexed to the α-(Fe,Si) phase by HRTEM images and corresponding Fourier transforms found in Figure 3e. Two types of shapes, such as the sphere and sheet of α-(Fe,Si), existed on the cooled surface of the ribbon during rapid solidification. The majority of the 1:13 phase, a matrix with equiaxed crystals of approximately 200 to 400 nm, is observed on the cooled surface with some spherical precipitations of α-(Fe,Si) (size, approximately 20 to 100 nm) as a minor phase as seen in the upper half of Figure 3a. The shape and density of the α-(Fe,Si) phase evolve along the white long arrows on the cooled surface from near to far from the copper wheel, in which the volume fraction of the fine spherical α-(Fe,Si) phase is found to decrease while that of the sheet-like α-(Fe,Si) phase increased. The spherical shape of the α-(Fe,Si) phase is replaced by the coarse irregular shape of the α-(Fe,Si) phase, and a higher density of the latter is precipitated when the ribbon surface is far from the cooper wheel (see short arrows and white triangles in Figure 3a). The corresponding selected area diffraction (SAED) pattern for the white triangle of Figure 3a is further identified as α-(Fe,Si) (Figure 3f). A higher degree of super-cooling gave rise to a nucleation rate of the 1:13 phase and the density and shape of the primary α-(Fe,Si) phase on the cooled surface of the ribbon.

**Figure 4:**
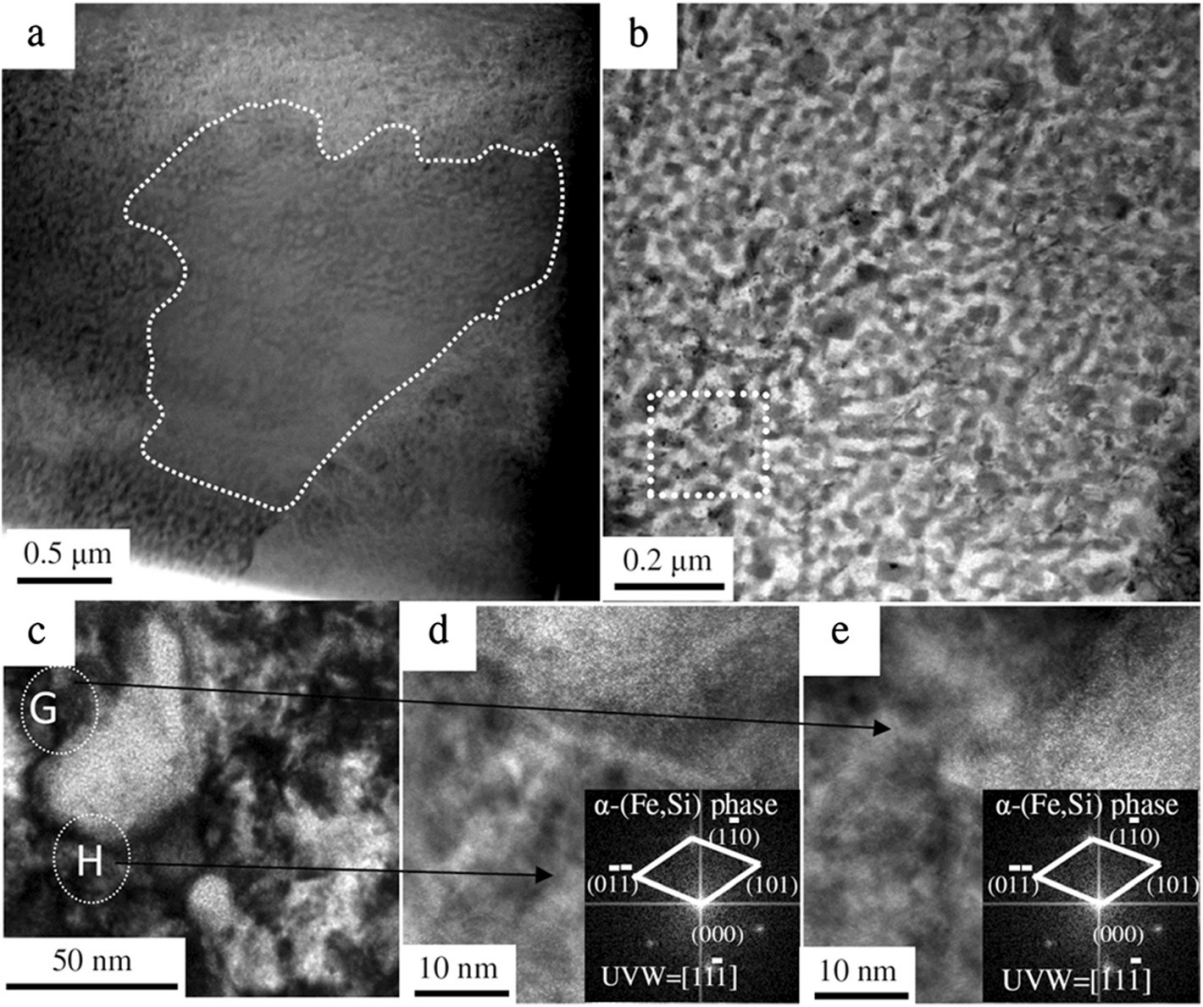
**The TEM micrographs results.** TEM micrographs of the free surface region for region B **(a)**, with higher magnifications of the ‘dashed region’ **(b)** and the ‘dashed box’ **(c)**. HRTEM images and corresponding FFT patterns of region G **(d)** and region H **(e)**.

Figure 4a shows a TEM micrograph of the free surface of the ribbon for region B with a magnification shown in Figure 4b. The microstructure consists of grain clusters, which were formed during the rapid solidification stage. The cluster boundary is well defined (see the white dashes in Figure 4a). Some nano-sized worm-like morphology was observed in the internal region of the clusters (Figure 4b). EDS revealed that the chemical composition of the black matrix was consistent with the α-(Fe,Si) phase (2.09 at.% La, 92.90 at.% Fe, and 5.00 at.% Si). The HRTEM images and corresponding Fourier transforms for the white circle of Figure 4c (regions ‘G’ and ‘H’) in Figure 4d and e can be indexed to the α-(Fe,Si) phase. The TEM analyses further confirm that the majority of the α-(Fe,Si) phase is in region B of Figure 2b, and the 1:13 phase as a majority is in region A of Figure 2b and c during rapid (melt-spinning) solidification process in La-Fe-Si alloys. These results are in good agreement with the XRD data and the simulated results using the classical nucleation theory. It is important to point out that in the melt spinning method, due to the enhanced ∆*T* in the cooled surface of a melt-spun La-Fe-Si ribbon, the desired 1:13 phase can be directly formed from the melt during melt-spinning. This clear understanding of the competitive nucleation mechanism between the 1:13 and α-(Fe,Si) phases allows us to address the emerging and important question of why the 1:13 phase does not form directly from the melt under equilibrium solidification conditions or under arc-melting, but from the rapid (melt spinning) solidification. It provides good guidance to the development of La-Fe-Si materials with desirable magnetic properties for a wide range of technological applications, such as magnetic refrigerant materials for use in active magnetic refrigerators.

## Conclusions

The nucleation mechanism involving rapid solidification of undercooled La-Fe-Si melts has been studied theoretically and experimentally. We find that for ∆*T* < 707 K, the α-(Fe,Si) phase has a higher nucleation rate compared to that of the 1:13 phase, and it is a primary phase in a slow solidification process. For ∆*T* > 707 K, the nucleation rate of the 1:13 phase is faster than that of the α-(Fe,Si) phase, resulting in a primary solidification of the 1:13 phase. As ∆*T* = 707 K, both of the 1:13 and α-(Fe,Si) phases have equal nucleation rates, but the microstructural morphology is distinctly different on the cooled and free surfaces of the ribbon. The desired nano-sized 1:13 phase can be directly formed from the melt during melt-spinning due to the enhanced ∆*T*.

## Abbreviations

EDS: energy dispersive spectrometer
GMCE: giant magnetocaloric effect
HRTEM: high resolution transmission electron microscope
SAED: selected area diffraction
SEM: scanning electron microscope
TEM: transmission electron microscope
XRD: x-ray diffraction
